# The role of YAP1 in survival prediction, immune modulation, and drug response: A pan-cancer perspective

**DOI:** 10.3389/fimmu.2022.1012173

**Published:** 2022-11-21

**Authors:** Xueqing Hu, Yingru Zhang, Hao Yu, Yiyang Zhao, Xiaoting Sun, Qi Li, Yan Wang

**Affiliations:** ^1^ Department of Medical Oncology, Shuguang Hospital, Shanghai University of Traditional Chinese Medicine, Shanghai, China; ^2^ Academy of Integrative Medicine, Shanghai University of Traditional Chinese Medicine, Shanghai, China

**Keywords:** *YAP1*, pan-cancer, immune, survival, drug response

## Abstract

**Introduction:**

Dysregulation of the Hippo signaling pathway has been implicated in multiple pathologies, including cancer, and *YAP1* is the major effector of the pathway. In this study, we assessed the role of *YAP1* in prognostic value, immunomodulation, and drug response from a pan-cancer perspective.

**Methods:**

We compared *YAP1* expression between normal and cancerous tissues and among different pathologic stages survival analysis and gene set enrichment analysis were performed. Additionally, we performed correlation analyses of *YAP1* expression with RNA modification-related gene expression, tumor mutation burden (TMB), microsatellite instability (MSI), immune checkpoint regulator expression, and infiltration of immune cells. Correlations between *YAP1* expression and IC_50_s (half-maximal inhibitory concentrations) of drugs in the CellMiner database were calculated.

**Results:**

We found that *YAP1* was aberrantly expressed in various cancer types and regulated by its DNA methylation and post-transcriptional modifications, particularly m6A methylation. High expression of *YAP1* was associated with poor survival outcomes in ACC, BLCA, LGG, LUAD, and PAAD. *YAP1* expression was negatively correlated with the infiltration of CD8+ T lymphocytes, CD4+ Th1 cells, T follicular helper cells, NKT cells, and activated NK cells, and positively correlated with the infiltration of myeloid-derived suppressor cells (MDSCs) and cancer-associated fibroblasts (CAFs) in pan-cancer. Higher *YAP1* expression showed upregulation of TGF-β signaling, Hedgehog signaling, and KRAS signaling. IC_50_s of FDA-approved chemotherapeutic drugs capable of inhibiting DNA synthesis, including teniposide, dacarbazine, and doxorubicin, as well as inhibitors of hypoxia-inducible factor, MCL-1, ribonucleotide reductase, and FASN in clinical trials were negatively correlated with *YAP1* expression.

**Discussion:**

In conclusion, *YAP1* is aberrantly expressed in various cancer types and regulated by its DNA methylation and post-transcriptional modifications. High expression of *YAP1* is associated with poor survival outcomes in certain cancer types. YAP1 may promote tumor progression through immunosuppression, particularly by suppressing the infiltration of CD8+ T lymphocytes, CD4+ Th1 cells, T follicular helper cells, NKT cells, and activated NK cells, as well as recruiting MDSCs and CAFs in pan-cancer. The tumor-promoting activity of *YAP1* is attributed to the activation of TGF-β, Hedgehog, and KRAS signaling pathways. AZD2858 and varlitinib might be effective in cancer patients with high *YAP1* expression.

## Introduction

The Hippo signaling pathway is an evolutionarily conserved pathway with a biological role in cell fate determination, organ size control, and tissue regeneration in most tissues and organs (1–4). Dysregulation of this pathway has been implicated in a variety of pathologies and has received extensive attention over the past two decades (5). In cancer research, the activated Hippo pathway is considered a tumor suppressor pathway due to its role in inhibiting cell proliferation and promoting apoptosis (6–8).

Growth factors, glucose, hypoxia, cell polarity, and mechanical cues from cell–cell or cell–extracellular matrix attachment regulate the Hippo pathway (9). The core components of the Hippo pathway in mammals consist of a kinase cascade, MST1/2 and LATS1/2, and the main effector YAP1/TAZ, a transcriptional coactivator without DNA-binding domains. The major binding partners of YAP1/TAZ are TEAD1–4. Other transcriptional factors, including AP1, PITX2, ZEB1, MYC, E2F, and SMADs, have also been reported to cooperate with the YAP1/TAZ–TEAD complex (10–15). In addition, RUNX2, TP73, and FOXO1 also directly bind to YAP1/TAZ (16). *YAP1* overexpression has been reported to be oncogenic in bile duct, breast, colon, lung, and liver cancers (17–23). Inhibition of the Hippo pathway or overexpression of *YAP1* may lead to the nuclear translocation of *YAP1*, which then binds to transcription factors to promote the expression of tumor-promoting genes. Of note, YAP1 also functions as a tumor corepressor to repress the expression of downstream genes, including the cell-cycle kinase inhibitor p27, by recruiting the NuRD (nucleosome remodeling deacetylase) complex, YY1, or EZH2, a polycomb repressive complex member (24, 25). Thus, the role of transcriptional cofactor or transcriptional corepressor of YAP1 is largely context-dependent. Therefore, better defining the role of *YAP1* in each cancer type will be a key challenge for future studies about target identification and cancer therapy. We investigated the potential role of *YAP1* in survival predication from a pan-cancer perspective.

Tumor-infiltrating immune cells are a major component of the ecosystem in tumor microenvironment (TME) and regulate tumor progression (26). Recently, the Hippo signaling pathway is emerging as an important pathway to affect immune function in cancer (27). *YAP1* has been reported to affect the activity of B cells, Tregs, macrophages, and myeloid-derived suppressor cells (MDSCs) in several cancer types (28). However, the role of *YAP1* in different cancer types and its mechanisms in immune regulation remain to be investigated. In this study, we analyzed the correlation between *YAP1* expression and infiltration of various immune cells in 33 cancer types.

Strategies to inhibit YAP1 activity include the following (1): Disrupting the YAP1–TEAD binding or blocking the transcriptional activity of the YAP1–TEAD complex. Carbonic anhydrase 3 (CA3) and verteporfin act by disrupting the YAP1–TEAD binding (29–31). The TDU domain of vestigial like family member 4 (VGLL4), a natural antagonist of YAP1, competes with YAP1 to bind TEADs (32). Narciclasine and peptide17 competes with TEAD4 for binding to YAP1 (33, 34). In addition, K-975, a TEAD inhibitor, inhibits YAP1/TAZ–TEAD interaction (35) (2). Targeting downstream targets of YAP1/TAZ (BCL-xL, FOXM1, and TG2). A37, celecoxib, TP-0903, cyclic peptide RA-V (deoxybouvardin), navitoclax, thiostrepton, and NC-9 fall into this category (36–43). Of these drugs, only verteporfin has been approved by Food and Drug Administration (FDA). However, *YAP1* may act in a TEAD-independent manner (44). The efficacy and selectivity of other drugs are not satisfactory. New drugs are needed to inhibit *YAP1* activity. In this study, we calculated the correlation between *YAP1* expression and IC_50_s of drugs with FDA approval or in clinical trials, which is a simple way to roughly assess the drug sensitivity.

In this study, we analyzed *YAP1* expression in 33 cancer types to reveal its role in predicating prognosis, modulating TME, and drug response to chemotherapeutic and targeted drugs that were FDA-approved or are in clinical trials.

## Methods and materials

### Data collection

Transcriptional RNA-sequence data [cohort: TCGA Pan-Cancer (PANCAN), batch effects normalized mRNA data], genome-wide DNA methylation levels (DNA Methylation 450K), and clinical characteristics (curated clinical data) of samples related to 33 cancer types were downloaded from UCSC Xena (https://xenabrowser.net/datapages/), which is derived from TCGA resources (45).

### Survival analysis

Survival information, including overall survival (OS), progression-free interval (PFI), disease-free interval (DFI), and disease-specific survival (DSS), was also downloaded from the UCSC Xena database. The Kaplan–Meier model and univariate Cox regression were then used to assess the prognostic value of *YAP1*. Bivariate *YAP1* expression levels were used to perform Kaplan–Meier curves analysis using the optimal cut point from the survminer R package (0.4.9) and survival R package (version 3.3.1). *p*-values of the Kaplan–Meier method and hazard ratio (HR) with a 95% confidence interval (95% CI) were calculated for each cancer type and presented as forest plots using the forestplot R package (version 2.0.1).

### Infiltration of immune cells

The proportions of 22 immune cell types and immune scores of all samples of 33 cancer types were downloaded from the supplementary data of the published paper (46). Infiltration of immune cells was performed with the CIBERSORT program, a method that uses gene expression profiles of complex tissues to calculate cell composition (47). The Xcell, TIMER, EPIC, quanTIseq, and MCP-counter programs were also used. TIMER provides the coefficients of six immune infiltrating cells indicating the relative abundance of immune cells (48). The EPIC program estimates the proportions of immune and cancer cells by separating the reference gene expression profiles of major non-malignant cell types (49). The ESTIMATE program was performed to infer tumor purity and immune cell admixture (immune score) from expression data (50). Pearson correlation coefficients between *YAP1* expression and infiltration of immune cells or immune score were calculated using the ggpubr R package (version 0.4.0), and scatter plots were visualized using ggplot2 (version 3.3.6).

### Gene set enrichment analysis

For each cancer type, samples with *YAP1* expression above the median level were grouped as high *YAP1* expression, and the others were grouped as low *YAP1* expression to compare the difference in their hallmark gene sets. Fifty hallmark gene sets were downloaded from the Molecular Signatures Database (MSigDB, https://www.gsea-msigdb.org/gsea/index.jsp), and gene set enrichment analysis (GSEA) was performed using GSEA software (version 4.2.3). One thousand times was set as the number of permutations and phenotypes was set as permutation type. Normalized enrichment score (NES) and nominal *p* value for each biological process were calculated for each cancer type. Hallmark gene sets with nominal *p* value < 0.05 were presented as a bubble plot using the ggplot2 R package.

### Correlation of *YAP1* expression with tumor mutation burden and microsatellite instability

Tumor mutation burden (TMB) andmicrosatellite instability (MSI) levels for each sample in pan-cancer were downloaded from the supplementary data of the published paper (46). Pearson correlation coefficients between *YAP1* expression and TMB or MSI levels for each cancer type were calculated using ggpubr R package and displayed as radar charts using fmsb R package (version 0.7.3).

### Azoxymethane/dextran sulfate sodium-induced colorectal cancer model and immunohistochemical staining of YAP1

CRC was induced in C57BL/6J mice by azoxymethane (AOM)/dextran sulfate sodium (DSS) as previously described (51). Colon sections of each mouse were collected for IHC staining and YAP1 was stained as previously described (51).

### Assessment of drug sensitivity

IC_50_s (half-maximal inhibitory concentrations) of drugs and gene expression of cancer cell lines were downloaded from the CellMiner database (https://discover.nci.nih.gov/cellminer/home.do). Only drugs with FDA approval or in clinical trials were included in further analyses. Pearson correlation coefficients between *YAP1* expression and the IC_50_ *z* score of each drug were calculated using the ggpubr R package and represented as bubble plots or scatter plots using the ggplot2 R package.

### Cell viability assay

Cells were seeded at a density of 1 × 10^4^ cells/well in 96-well plates. When cells reached 60% confluence, drugs were added into the wells and incubated for 24 h or 48 h. Medium containing 10% Cell Counting Kit-8 (CCK-8) reagent (Dojindo, Japan) was then added into the cells and incubated for another 1.5 h at 37°C. The light absorbance was measured at 450 nm on the microplate reader (Bio-Rad, USA). Each group was performed in sextuplicate. Verteporfin, AZD-2858 were purchased from TargetMol, USA, and varlitinib was purchased from MCE, USA

### Western blot assay

Cellular proteins were extracted with protein extraction reagent (Beyotime Biotechnology) and quantified by BCA protein assay (Beyotime Biotechnology). A total of 12 μg of protein per sample was added to SDS-PAGE gels for electrophoresis (100 V, 2 h), followed by constant flow membrane transfer (ice bath, 210 mA, 2 h). The transferred polyvinylidene fluoride (PVDF) membranes were blocked with 5% BSA-containing Tris-buffered saline with Tween (TBST) for 2 h at room temperature, and incubated with primary antibodies overnight at 4°C. Then, membranes were washed three times with TBST and incubated with secondary antibody at room temperature for 2 h. Membranes were examined with a gel imager (ECL, Millipore, USA). Antibodies of Vimentin, E-Cadherin, smad2, ERK, p-ERK, CREB, and GAPDH were purchased from Cell Signaling Technology (CST); YAP1, p-YAP1, and PD-L1 were purchased from Proteintech. All primary antibodies were used at 1:1,000 dilution. Secondary antibodies were purchased from Beyotime Biotechnology (1:2,000 dilution).

### Reverse transcription-quantitative polymerase chain reaction

Primers were designed using the Primer-Blast tool at NCBI (https://www.ncbi.nlm.nih.gov/tools/primer-blast/) and synthesized by Sangon Biological Engineering Co., Ltd. (Shanghai, China). The sequences of the primers are listed in **Supplementary Table S1**. Total cellular RNA was extracted with RNA Trizol (Beyotime Biotechnology). Reverse transcription of RNA was performed using HiScript III RT SuperMix (Vazyme). The SYBR qPCR Mix (Vazyme) was used in a 10-µl reaction mixture that included 1 µl of cDNA template, 0.4 µl of each 0.5 µM primer, 3.6 µl of ddH_2_O, and 5 µl of 2× SYBR buffer. The reaction was performed with 1 cycle of 30 s at 95°C and 50 cycles of 10 s at 95°C, 30 s at 60°C, and 15 s at 60°C. Actin was used as the reference mRNA. The qPCR reaction was performed in triplicate.

### Statistical analysis

Differences in *YAP1* mRNA expression between normal and cancer tissues or between the two pathological stages of each cancer type were tested by Wilcox test. The differences in the proportions of the four pathological stages between groups with high or low *YAP1* expression were compared by chi-squared test. Differences in OS, PFI, DFI, and DSS between those two subgroups were compared using the Kaplan–Meier method and log-rank rest. The HRs were calculated by univariate Cox regression. All *p* values were two-sided, and *p* < 0.05 was considered statistically significant.

## Results

### *YAP1* is aberrantly expressed in various cancer tissues

**Figure 1:**
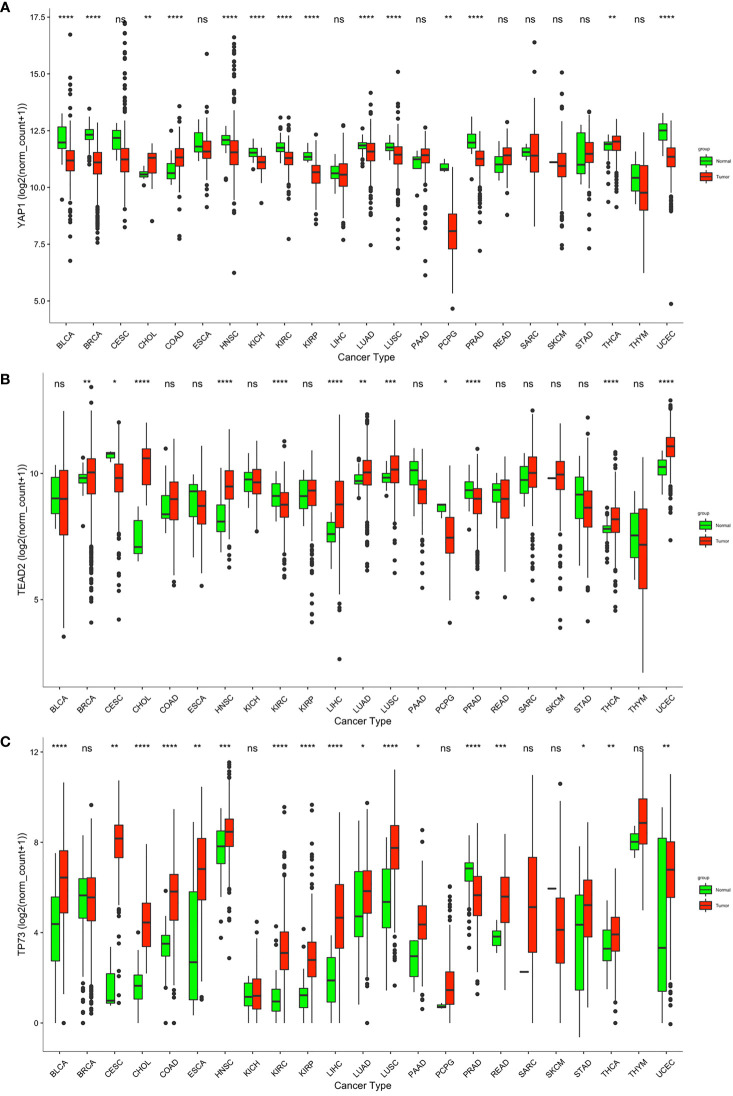
Transcriptional level of YAP1, TEAD2, and TP53 in normal and cancer tissues according to RNA-sequence data from TCGA. ****p < 0.0001, ***p < 0.001, **p < 0.01, *p < 0.05, and ns p > 0.05.

We compared *YAP1* mRNA expression between normal and primary cancer tissues in 23 cancer types. For the other 10 cancer types, RNA-sequence data from paired normal tissues were not available. *YAP1* was differently expressed in 14 of the 23 cancer types with statistical significance. Among them, *YAP1* expression was upregulated in cholangiocarcinoma (CHOL), colon adenocarcinoma (COAD), and thyroid carcinoma (THCA), and was downregulated in bladder urothelial carcinoma (BLCA), breast invasive carcinoma (BRCA), head and neck squamous cell carcinoma (HNSC), kidney chromophobe (KICH), kidney renal clear cell carcinoma (KIRC), kidney renal papillary cell carcinoma (KIRP), lung adenocarcinoma (LUAD), lung squamous cell carcinoma (LUSC), pheochromocytoma and paraganglioma (PCPG), prostate adenocarcinoma (PRAD), and uterine corpus endometrial carcinoma (UCEC) (**Figure 1A**). IHC staining also identified that *YAP1* was upregulated in colon cancer tissues versus normal colonic mucosa tissues (52). *YAP1* has been reported to be upregulated in CHOL and THCA (17, 18). *YAP1* does not contain DNA-binding sequences; thus, the binding partners are important for its function. *YAP1* binds to TEADs to facilitate the expression of tumor-promoting genes, and YAP1 may switch to bind to TP73 to promote apoptosis of cancer cells (19). Therefore, we also analyzed the expression of *TEADs* and *TP73* in pan-cancer. Consistent with *YAP1* expression, *TEAD2* was upregulated not only in CHOL, COAD, and THCA, but also in BRCA, HNSC, liver hepatocellular carcinoma (LIHC), LUAD, LUSC, and UCEC (**Figure 1B**). Expression of *TEAD1/2/4* is shown in **Supplementary Figure S1**. TP73 was also upregulated in most cancer types, including CHOL, COAD, and THCA (**Figure 1C**). Xia et al. reported that high levels of *YAP1* expression were positively correlated with *TEAD4* gene expression, and their co-expression was a prognostic marker for poor ovarian cancer survival (53). High expressions of *YAP1* and *TEADs* and their target genes were associated with low OS in patients with non-metastatic human gastric carcinomas (54). Strano et al. reported that physical interaction with YAP1 protein enhanced transcriptional activity of TP73 (55). High expression of *YAP1, TEAD4*, and *TP73* was significantly associated with high grade, advanced stage, supraglottic location of tumors, nodal metastases, and recurrence of human laryngeal cancer. In addition, high expression of all proteins was significantly associated with poor overall and disease-free survival (56). The tumor-promoting or tumor-suppressing role of the YAP1–TP73 complex, as well as the binding preference mechanism of YAP1 remains to be revealed.

### *YAP1* expression correlates with its DNA methylation and RNA modification

**Figure 2:**
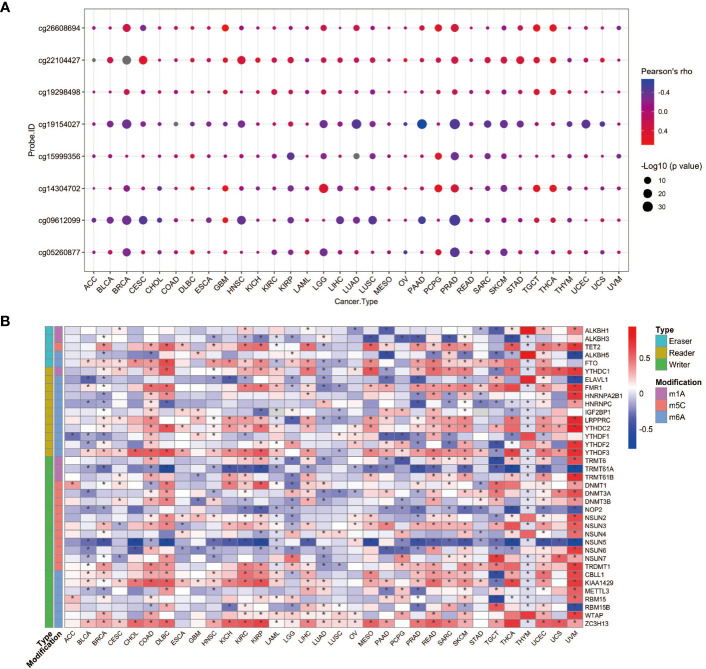
YAP1 expression correlates with its DNA methylation and RNA modification. **(A)** Pearson correlation between YAP1 expression and its DNA methylation in 33 cancer types. **(B)** Pearson correlation between YAP1 expression and RNA modification regulators. *p < 0.05.

To seek the potential regulation of *YAP1* expression by DNA methylation and post-transcriptional RNA modifications, we performed correlation analyses. DNA methylation levels of eight CpG sites in the 5’UTR of *YAP1* were included. In general, *YAP1* expression was negatively correlated with its DNA methylation level in most cancer types, suggesting that DNA demethylation in the 5’UTR of *YAP1* may promote its expression (**Figure 2A**). RNA modifications play pivotal roles in RNA stability and translation efficiency. We found that a wide range of RNA modification-related genes were positively correlated with *YAP1* expression. Genes responsible for reading, writing, and erasing the modifications on m1A, m5C, and m6A were broadly associated with *YAP1* expression in all cancer types, particularly in lymphoid neoplasm diffuse large B-cell lymphoma (DLBC), rectum adenocarcinoma (READ), skin cutaneous melanoma (SKCM), uveal melanoma (UVM), and testicular germ cell tumors (TGCT) (**Figure 2B**). Among the 37 regulators, RNA m6A methylation readers and writers, including *ZC3H13, LRPPPRC, YTHDC2, YTHDF3*, and *KIAA1429*, were positively correlated with *YAP1* expression in more than 20 of the 33 cancer types with statistical significance, suggesting the potential role of RNA m6A methylation in facilitating *YAP1* mRNA stability.

### Clinical prognostic value of *YAP1* in pan-cancer

**Figure 3:**
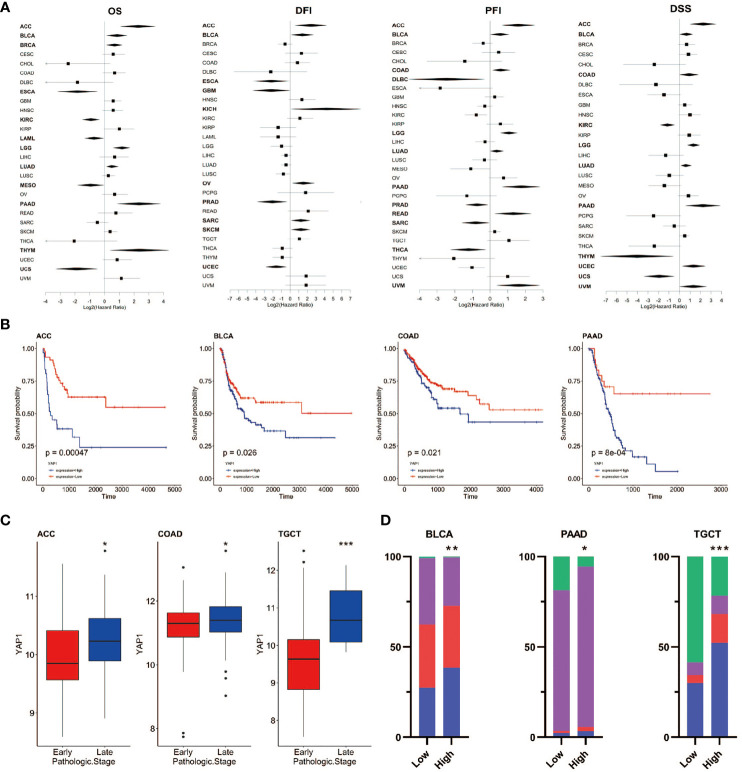
Prognostic value of YAP1 expression in pan-cancer. **(A)** Survival outcomes of cancer patients with high or low YAP1 expression. Bold lines: p < 0.05. **(B)** Survival curves of PFI in ACC, BLCA, COAD, and PAAD. **(C)**YAP1 expression between patients with the early or late pathologic stages of cancer. **(D)** Distribution of pathologic stages in BLCA, PAAD, and TGCT. ***p < 0.001, **p < 0.01, and *p < 0.05.

To assess the role of *YAP1* in predicating clinical outcomes of patients in 33 cancer types, we analyzed OS, DSS, DFI, and PFI using the Kaplan–Meier method (log-rank test) and univariate Cox regression. High *YAP1* expression was a risk factor for the OS in seven cancer types, PFI in eight cancer types, DFI in six cancer types, and DSS in eight cancer types. In general, high *YAP1* expression was risky in adrenocortical carcinoma (ACC), BLCA, COAD, brain lower grade glioma (LGG), LUAD, and pancreatic adenocarcinoma (PAAD), but it is a protective factor in esophageal carcinoma (ESCA), KIRC, PRAD, and mesothelioma (MESO) (**Figures 3A, B**). Of note, high *YAP1* expression was a risk factor for all four prognostic survival indicators of ACC and BLCA (**Figure 2A**). Therefore, the role of *YAP1* is largely context-dependent. In ACC, COAD, and TGCT, *YAP1* expression was higher in the late pathologic stage (AJCC pathologic stages III and IV) than that in the early stage (AJCC pathologic stages I and II) (**Figure 3C**). In BLCA, PAAD, and TGCT, a higher frequency of patients with more severe stages was observed in patients with higher *YAP1* expression compared with those with lower *YAP1* expression (**Figure 3D**). Those results demonstrate that *YAP1* may promote cancer progression in ACC, COAD, PAAD, and TGCT. Among them, the tumor-promoting role of *YAP1* in COAD and PAAD has been determined experimentally (57, 58).

### *YAP1* induces immunosuppressive TME

**Figure 4:**
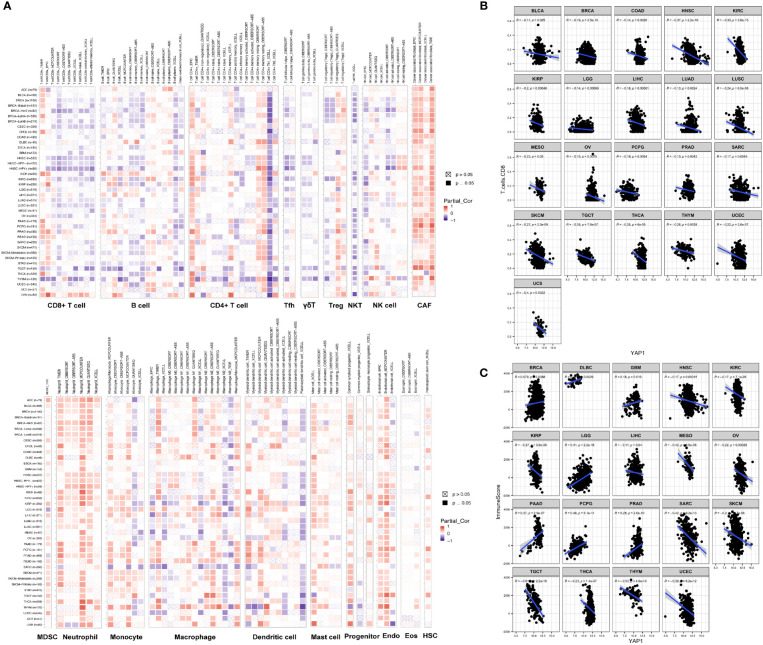
Correlation of *YAP1* expression with immune cell infiltration. **(A)** Pearson correlation between *YAP1* expression and infiltration of 22 immune cell types using different programs. Positive correlation is in red and negative correlation is in blue. *p < 0.05. **(B)** Pearson correlation between *YAP1* expression and infiltration of CD8+ T cells in pan-cancer. Only statistically significant cancer types were shown. **(C)** Pearson correlation between *YAP1* expression and immune score. Only statistically significant cancer types were shown. Tfh, T follicular helper cells. Treg, regulatory T cells; NKT, natural killer T cells; NK, natural killer cells; CAF, cancer-associated fibroblast; MDSC, myeloid-derived suppressor cell; Endo, endothelial cell; Eos, eosinophil; HSC, hematopoietic stem cell. γδ T cells, gamma-delta T cells.

TME contains tumor cells, immune cells, and stromal cells, which play pivotal roles in cancer initiation, progression, and drug response. We analyzed the correlation between *YAP1* expression and infiltration of 19 cell types in tumor tissues using CIBERSORT, Xcell, TIMER, EPIC, quanTIseq, and MCP-counter programs. We found that *YAP1* expression was associated with infiltration of cells in both innate and adaptive immune system in pan-cancer. In general, *YAP1* expression was negatively correlated with the infiltration of CD8+ T cells, T follicular helper cells, γδ T cells, activated natural killer (NK) T cells, CD4+ Th1 cells, and myeloid dendritic cells in most cancer types, but positively correlated with the infiltration of MDSCs, cancer-associated fibroblasts (CAFs), and neutrophil cells (**Figure 4A**). These results were consistent using different tools, except for the filtration of CD8+ T cells calculated by TIMER and other programs. EPIC and TIMER programs showed an opposite correlation between *YAP1* expression and infiltration of CD4+ T cells in a few cancer types. The correlation of *YAP1* expression with infiltration of regulatory T cells (Tregs), plasma B cells, NK cells, and macrophage was contradictory when using different programs (**Figure 4A**). Thus, experiments are needed to confirm the results. Notably, CD8+ T cells are a key subset of MHC class I-restricted T cells and are one of the major mediators of adaptive immunity. High expression of *YAP1* may inhibit the infiltration of CD8+ T cells in 21 of the 33 cancer types (BLCA, BRCA, COAD, HNSC, KIRC, KIRP, LGG, LIHC, LUAD, LUSC, OV, MESO, PCPG, PRAD, SARC, SKCM, TGCT, THCA, THYM, UCEC, and UCS), indicating its immunosuppressive role in TME (**Figure 4B**). Immune score predicts the level of infiltrating immune cells. *YAP1* expression was negatively with the immune score in 12 cancer types (HNSC, KIRC, KIRP, LIHC, MESO, OV, SARC, SKCM, TGCT, THCA, THYM, and UCEC). However, it was positively correlated with the immune score in BRCA, DLBC, GBM, LGG, PAAD, PCPG and PRAD (**Figure 4C**). *IL-6, CSF-1, CSF-2, CSF-3*, and *CXCL5*, which are capable of recruiting MDSCs, were downregulated in CRC cells when YAP1 was inhibited by verteporfin (**Figure 8C**).

### Correlations between *YAP1* and immune checkpoints, TMB, and MSI

**Figure 5:**
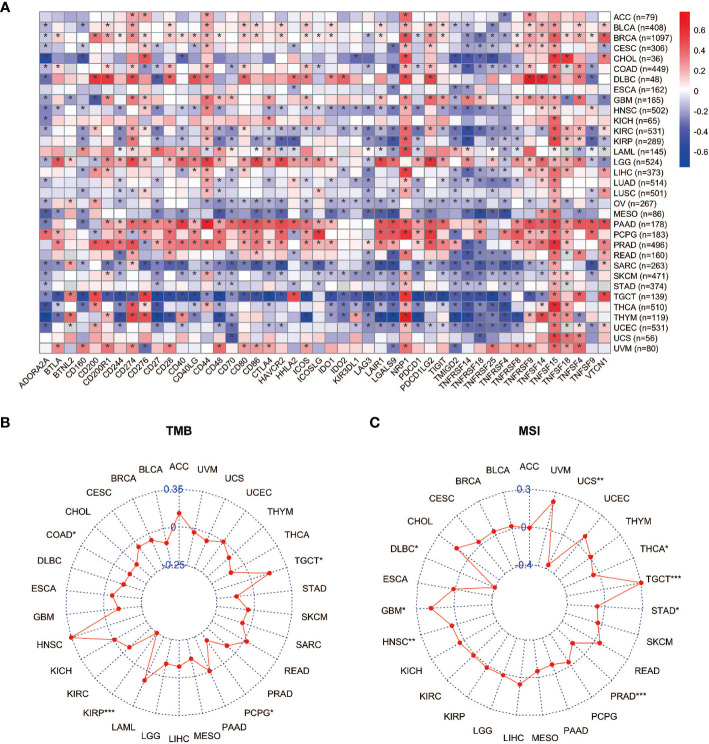
Correlation between expression of *YAP1* and immune checkpoints, TMB, and MSI. **(A)** Heat map of correlation between *YAP1* expression and 46 immune checkpoint regulators. *p < 0.05. **(B, C)** Correlation between *YAP1* expression, TMB, and MSI. ***p < 0.001, **p < 0.01, and *p < 0.05.

Immune checkpoints are regulators of the immune system. They consist of a group of programmed death receptors and their ligands expressed on immune cells. Tumor cells can evade immune destruction by upregulating immune checkpoints (59). Therefore, we performed the Pearson correlation analyses to reveal the relationship between *YAP1* expression and 46 immune checkpoint regulators. We found that *YAP1* expression was positively correlated with most immune checkpoint genes and immune cell marker genes in all 33 cancer types. *CTLA-4, TIM-3 (HAVCR2)*, and *PD-1 (PDCD1)* are key checkpoint regulators that suppress immune response. They all positively correlated with *YAP1* expression in LGG, PAAD, and PRAD, but negatively correlated with *YAP1* expression in MESO, SARC, TGCT, and UCEC (**Figure 5A**). We confirmed that YAP1 inhibitor verteporfin greatly reduced PD-L1 expression in CRC cell lines (**Figure 8B**). In most cancer types, *CD274 (PD-L1), NRP1*, and *TNFSF15* were positively correlated with *YAP1* expression. In PAAD, PRAD and PCPG, *YAP1* expression was highly correlated with the expression of most immune checkpoint genes, suggesting that YAP1 may help cancer cells to evade immune destruction (**Figure 5A**). TMB refers to the total number of mutations per million bases (60). It is considered a promising biomarker of immune response, as tumors with high mutations are associated with high tumor neo-antigen burden, making them immunogenic, and therefore being more responsive to immunotherapy (61–64). MSI is a condition of genetic susceptibility to mutation due to impaired DNA mismatch repair (65). It is also used as a major predictive marker for the efficacy of immune checkpoint blockade therapies (66). We found that *YAP1* expression was positively correlated with TMB in TGCT and was negatively correlated in PCPG, KIRP, and COAD (**Figure 5B**). In addition, *YAP1* expression was positively correlated with MSI in GBM, HNSC, and TGCT and was negatively correlated with MSI in DLBC, PRAD, THCA, and UCS (**Figure 5C**). Our results suggest that *YAP1* may predict the efficacy of immune checkpoint inhibitors in TGCT, GBM, and HNSC.

### *YAP1*-associated cancer hallmarks

**Figure 6:**
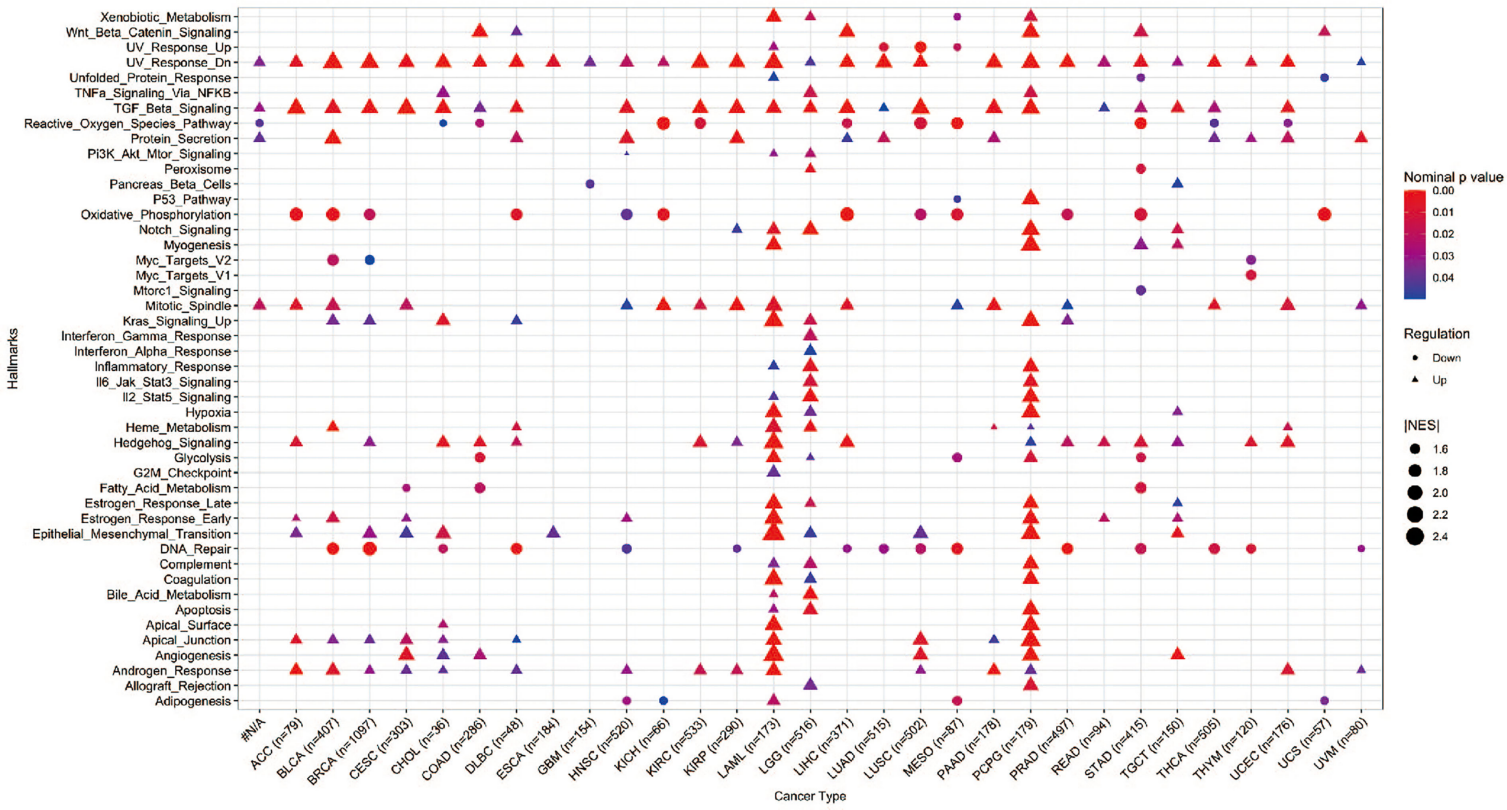
GSEA of hallmarks in cancer patients with high *YAP1* expression versus those with low YAP1 expression. Only statistically significant hallmarks (p < 0.05) were shown.

Hallmark gene sets summarize and represent specific well-defined biological states or processes and display coherent expression. We subdivided patients into two groups for each cancer type based on *YAP1* expression above or below the median level, and analyzed the differences in 50 hallmark gene sets across 33 cancer types. More than 23 hallmarks were aberrantly upregulated in LAML, LGG, and PCPG patients with high *YAP1* expression versus those with low *YAP1* expression. Various hallmark gene sets were upregulated in pan-cancer. TGF-β signaling pathway was upregulated in 23 cancer types, mitotic spindle process in 16 cancer types, Hedgehog signaling pathway in 15 cancer types, KRAS signaling in 8 cancer types, epithelial mesenchymal transition (EMT) in 10 cancer types, and angiogenesis in 7 cancer types, indicating that *YAP1* is involved in promoting cell proliferation, cancer cell stemness, invasiveness, and migration processes (**Figure 6**). We then confirmed these results on CRC cell lines (Lovo and SW620). As expected, YAP1 was upregulated in AOM/DSS-induced colitis-associated cancer in C57BL/6J mice (**Figure 8A**). The YAP1 inhibitor verteporfin significantly reduced their cell viability (**Figure 8B**). Verteporfin also reduced expression of p-ERK (KRAS signaling pathway) and smad2 (TGF-β signaling pathway), and downregulated Wnt target genes *AXIN2*, *BIRC5, CCND1*, and *CD44* (**Figures 8C, D**). In addition, upregulation of E-cadherin (epithelial maker) and downregulation of vimentin (mesenchymal marker) were observed in verteporfin treatment, suggesting a role of YAP1 in promoting EMT (**Figure 8D**). Of note, those results were based on enrolled cancerous samples. The hallmark difference between samples with higher and lower *YAP1* expression is less than that between the samples with or without *YAP1* function. Thus, the YAP1-associated hallmarks included but were not limited to those mentioned above

### Potential drugs for *YAP1*

**Figure 7:**
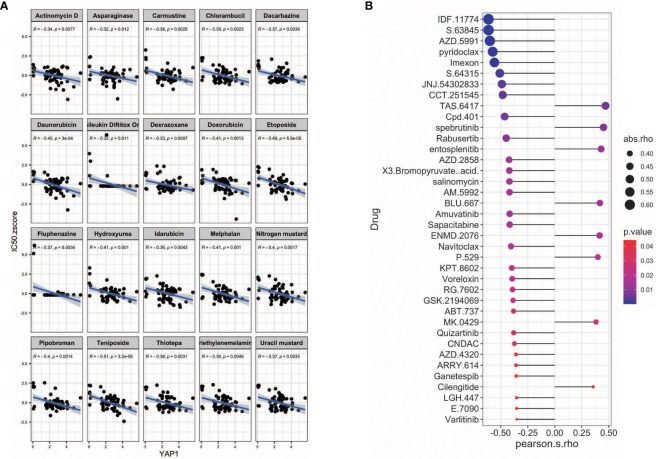
Potential drugs for *YAP1*. **(A)** Correlation of *YAP1* expression with and IC_50_ *z* score of FDA-approved drugs (top 25). **(B)** Correlation of *YAP1* expression and IC_50_ of drugs in clinical trial.

**Figure 8:**
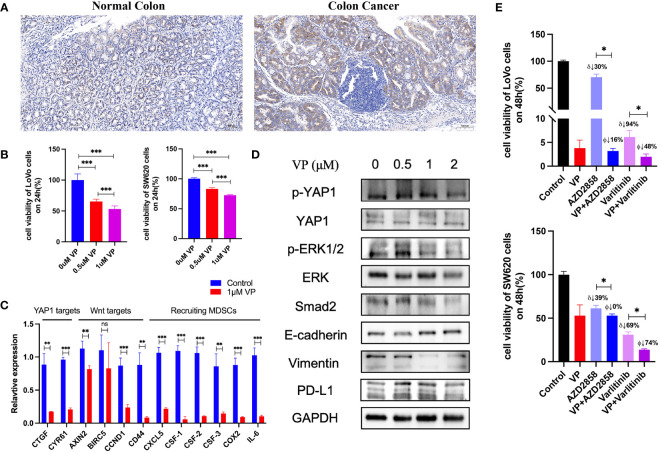
Experiments to confirm the activities of *YAP1*. **(A)** IHC staining of YAP1 in normal colon tissue and AOM/DSS-induced colitis-associated CRC in C57BL/6J mice. **(B)** Effect of YAP1 inhibitor verteporfin (VP) on the viability of CRC cells (24 h). **(C)** Regulation of YAP1 inhibitor on Wnt target genes and chemokines that recruit MDSCs’ infiltration in LoVo cell line. **(D)** YAP1 is involved in KRAS and TGF-β signaling pathways as well as EMT processes in Lovo cell line. **(E)** Effect of AZD2858 and varlitinib in combination with YAP1 inhibitor on the viability of CRC cells. δ: versus control group, ϕ versus verteporfin group. VP: 1 μM; AZD2858: 1 μM; varlitinib: 5 mM. ns (no significance), *p < 0.05, **p < 0.01, and ***p < 0.001.

We further screened potential drugs that may be effective in cancer patients with high *YAP1* expression. We downloaded the *z* scores of IC_50_s of 24,360 drugs in 60 cancer cell lines. Only 218 FDA-approved drugs and 574 drugs in clinical trials were included for correlation analysis. Of the 218 FDA-approved drugs, chemotherapeutic agents that inhibit DNA synthesis (teniposide, dacarbazine, doxorubicin, triethylenemelamine, nitrogen mustard, etoposide, and thiotepa) topped the list, which might be effective in treating cancer patients with high *YAP1* expression. Interestingly, an antipsychotic medication, fluphenazine, may also have an effect. Inhibitors of hypoxia-inducible factor (IDF-11774), MCL-1 (S63845, AZD-5991, pyridoclax, and S-64315), ribonucleotide reductase (imexon), FASN (JNJ-54302833), WNT signaling (CCT251545), STAT/STAT3 (CPD-401), and CHK (rabusertib) were among the top 10 drugs in clinical trial that may work in patients with high *YAP1* expression, whereas inhibitors of EGFR (TAS6417), Bruton’s agammaglobulinemia tyrosine kinase (BTK) (spebrutinib), SYK (entospletinib), RET (Blu667), VEGFR2 (ENMD-2076, P-529), and αvβ3 integrin (MK-0429, cilengitide) may not work (**Figure 7**). We then treated Lovo and SW620 cell lines with AZD2858 and varlitinib alone or in combination with verteporfin. These results showed that the inhibitory rate of combined treatment *versus* verteporfin is smaller than that of AZD2858 or varlitinib alone *versus* control, indicating that the role of AZD2858 and varlitinib in cytotoxicity was partially dependent on YAP1 activity (**Figure 8E**). In addition, the combination of varlitinib and verteporfin was more effective than either single agent. KRAS is an effector molecule responsible for signal transduction from ligand-bound EGFR to the nucleus. Varlitinib is a targeted drug against EGFR. Dual inhibition of EGFR and YAP1 obtained better therapeutic outcomes, suggesting that the KRAS pathway was involved in YAP1 activity, as we analyzed *in silico* (**Figure 6**).

## Discussion

*YAP1* is a transcriptional co-activator and a major effector of the Hippo signaling pathway. Emerging work indicates that *YAP1* is widely activated in human malignancies and is essential for cancer initiation, progression, and drug resistance in most solid tumors. High expression of *YAP1* has been reported to promote the excessive cell proliferation in multiple tissues, including liver, gastrointestinal tissue, skin, and heart (67–70). TEAD-dependent *YAP1* function has also been linked to invasive and metastatic behavior of tumor cells (71). Compelling evidence also showed that *YAP1* can alter the TME by recruiting immunosuppressive cell types, suppressing cytotoxic T-cell function or promoting the polarization of tumor-associated macrophages towards the pro-tumor M2 phenotype (72–74). In this study, we analyzed *YAP1* expression in pan-cancer and evaluated its role in prognostic value, immunomodulation, and drug response.

According to the TCGA database, *YAP1* expression was elevated in CHOL, COAD, and THCA, but decreased in 11 of the 23 cancer types, namely, BLCA, BRCA, HNSC, KICH, KIRC, KIRP, LUAD, LUSC, PCPG, PRAD, and UCEC (**Figure 1**). However, *YAP1* has been reported to be oncogenic in BLCA (75, 76), BRCA (77), HNSC (78), KICH (79), KIRC (80), KIRP (81), LUAD (82), LUSC (83), PRAD (84), and UCEC (85). PCPG is a rare adrenal tumor. The role of *YAP1* in PCPG remains to be investigated. The downregulation of *YAP1* in those caners might be due to case number limitation, and in addition to *YAP1* expression level, *YAP1* activity also depends on its cellular location. High *YAP1* expression is a risk factor for survival outcomes in ACC, BLCA, LGG, LUAD, PAAD, and COAD (**Figure 3**). Moreover, in ACC, BLCA, COAD, and TGCT, *YAP1* was associated with more severe pathologic stages (**Figure 3**). Among them, the prognostic value of *YAP1* on BLCA (84), LUAD (86), PAAD (87) and COAD (88) has been reported.

According to our result, high expression of *YAP1* predicted poor survival outcomes in patients with LUAD, COAD, and PAAD, and was associated with more severe pathologic stages in patients with COAD, PAAD, and TGCT (**Figure 3**). PAAD has been reported to be characterized by immunosuppressive TME. In this study, *YAP1* in PAAD was positively correlated with a wide range of immune checkpoint regulators. *YAP1* expression was positively correlated with levels of TMB and MSI in TGCT. In addition, important immune cells, including activated CD4+ memory T cells and T follicular helper cells, were positively correlated with *YAP1* expression in LUAD. Therefore, we would like to discuss in more detail the role of *YAP1* in these cancer types.

Elevated expression of the gene signature for *YAP1/TAZ* activity is associated with poor prognosis in patients with non-small-cell lung cancer (NSCLC) (89, 90), which is consistent with the results of LUAD in our study (**Figure 3**). Our GSEA results suggested that high *YAP1* expression was associated with reduced activities of DNA repair processes (**Figure 6**), and defective DNA repair contributes to individual susceptibility to lung cancer development (91). We also showed that TGF-β signaling was upregulated in LUAD with high *YAP1* expression (**Figure 6**). TGF-β is the most potent inducer of EMT in NSCLC and is pivotal to the development of tumor-promoting microenvironment in the lung cancer tissues (92). In our findings, *YAP1* may inhibit infiltration of CD8+ T cells, CD4+ Th1 cells, T follicular helper cells, and NKT cells, but increase infiltration of CAFs, which promote the development of LUAD. Zhang et al. have reported that *YAP1* activation was not sufficient to trigger NSCLC formation, but promoted its progression to higher grades (89, 93). The role of *YAP1* in immune cell infiltration in LUAD will be further experimentally confirmed in our future studies.

High level of *YAP* activity has been found to be prognostic for poor outcome in four datasets of CRC patients (94). In our study, *YAP1* expression was associated with severe pathologic stage and poor survival outcome in COAD (**Figure 3**). *YAP1/TAZ* is required for the formation and growth of intestinal tumors (95). Among the enriched hallmarks, higher *YAP1* expression was associated with upregulated Wnt β-catenin signaling, TGF-β signaling *via* NFKB, and Hedgehog signaling (**Figure 6**). Overactivation of the Wnt signaling pathway is the most oncogenic pathway in CRC. In addition, β-catenin-driven cancers require the *YAP1* transcriptional complex for tumorigenesis (96), suggesting the network between the two signaling pathways. Barry et al. reported that forced *YAP1* overexpression in the gut epithelium was not sufficient to form tumor (95). The lack of Wnt signaling pathway might be the reason. In COAD, *YAP1* expression was positively correlated with the infiltration of MDSCs and CAFs, and was negatively correlated with the infiltration of CD8+ T cells, CD4+ Th1 cells, activated NK cells, and NKT cells (**Figure 4**). Mechanically, *YAP1* promotes MDSC induction by inhibiting *PTEN* expression, resulting in upregulation of *COX-2, p-AKT*, and *p-p65* in CRC-derived cells, which leads to secretion of the cytokine granulocyte-macrophage colony-stimulating factor (97). *YAP1*-dependent matrix remodeling is required for the generation and maintenance of CAFs (98). Mechanisms of *YAP1* in recruiting CAFs and suppressing CD4+ Th1 cells, T follicular helper cells, activated NK cells, and NKT cells are yet to be unraveled.

PAAD is a very aggressive neoplasia that seems to arise from pancreatic exocrine cells. Pancreas-specific *YAP1* knockout halted tumor progression (99). Activation of *YAP1* in acinar cells upregulated JAK-STAT3 signaling and promoted the development of pancreatic cancer (100). In this study, *YAP1* expression was higher in PAAD patients with more severe pathological stages (**Figure 3**). Mechanically, *YAP1* and the ZEB1 complex activates *ITGA3* to promote its metastasis (101). PAAD is characterized by immunosuppressive TME. In our results, *YAP1* expression was positively correlated with MDSC infiltration and negatively correlated with NKT cells. Mielgo et al. reported that *YAP1* recruited MDSCs to suppress T-cell function and generate an immunosuppressive microenvironment (102). The mechanism remains to be clarified.

Testicular cancer is a very common malignancy in young men. Although testicular cancer has a high cure rate, patients have a high long-term risk of secondary malignant tumors. Ji et al. have reported that different immune status in TME may be responsible for the different survival outcomes of TGCT patients (103). Moreover, YAP1 inhibition enhanced the chemosensitivity of cisplatin in TGCT (104). Studies about the YAP1 in immunomodulation in TGCT remain absent.

In this study, from a pan-cancer perspective, high expression of *YAP1* was associated with upregulation of TGF-β signaling, KRAS signaling, Hedgehog signaling, EMT, and androgen response in most cancer types (**Figure 6**). There was a close interplay between YAP1 and TGF-β signaling. *YAP1* activation promoted TGF-β expression, which was involved in the biological processes of endothelial-to-mesenchymal transition (EMT) and fibrosis (105–107) in the liver, lung, and kidney. Their interactions in other organ tissues need to be investigated. We confirmed that YAP1 was able to upregulate smad2 in TGF-β signaling in CRC cell lines (**Figure 8**). KRAS and YAP1 converged on the transcription factor FOS and activated a transcriptional program involved in regulating EMT (108). However, another study found limited overlap of gene expression between KRAS G12V and YAP1 S127A-driven tumors (109). The interplay between YAP1 and KRAS signaling remains largely unknown. In this study, YAP1 inhibitor reduced the KRAS pathway activity in CRC (**Figure 8**). Hedgehog signaling activation could upregulate *YAP1* expression and induce osteosarcoma development (110), or aid in generation of liver (111). The cooperation between Hedgehog and YAP1 signaling in tumor formation and progression remains to be uncovered*. YAP1* mRNA was upregulated in androgen-insensitive prostate cancer cells (112), but the mechanism of their interaction is largely unknown. Furthermore, in our findings, the YAP1 inhibitor did reduce EMT markers in CRC.

Emerging evidence demonstrated the role of *YAP1* in modulating TME. In this study, we found that *YAP1* may suppress the infiltration of CD8+ T lymphocytes, γδ T cells, T follicular helper cells, NKT cells, and activated NK cells. Moreover, *YAP1* may recruit the CAFs and MDSCs to tumor site to suppress the immune response. Among them, the relationship between *YAP1* and infiltration of CD8+ T lymphocytes, MDSCs, and CAFs has been reported in several cancer types. However, the mechanisms are incomplete. The role of *YAP1* in the infiltration and activities of T follicular helper cells, NKT cells, and activated NK cells remains to be investigated. Stampouloglou et al. reported that *YAP1* overexpression in T cells reduced their activation, differentiation, and function, which translated *in vivo* into an impairment of T-cell infiltration and tumor repression (113). Another study confirmed that *YAP1* attenuated CD8+ T cell-mediated anti-tumor response (114). Mechanically, *YAP1* overexpression in cancer cells could upregulate *PD-L1* expression and impede the activities of CD8+ T cells in melanoma (115). Another mechanism is that YAP1/TEAD directly upregulates *CXCL5* in cancer cells to recruit CXCR2-expressing MDSCs, leading to decreased infiltration of CD8+ T cells (74). In addition, *IL-6* and *CSF1-3* induced by *YAP1* in PAAD stimulated the accumulation of MDSCs (116), and upregulation of *COX2* by *YAP1* in human granulosa cells promoted the recruitment of MDSCs (117). The role of *YAP1* in CD8+ T cells and MDSCs in other cancer types remains to be unraveled. In this study, we confirmed the results by experiments on CRC cells and found that the YAP1 inhibitor verteporfin decreased the expression of *PD-L1, CXCL5, COX-2, IL-6*, and *CSF1-3* (**Figure 8**). Mechanotransduction-mediated *YAP1* activation establishes a feed-forward self-reinforcing loop that contributes to maintenance of the CAF phenotype and promotes breast cancer invasion (98). Mechanistically, active *YAP1* promotes the expression of *ANLN* and *DIAPH3* and stabilizes actomyosin proteins, which is required for the generation and maintenance of CAFs (98). Another mechanism is that high expression of *YAP1* in the tumor stromal cells converts normal fibroblasts into CAFs in the TME of prostate cancer (118). The function of *YAP1* in CAFs of other cancer types remains largely unknown. In this study, high expression of *YAP1* was associated with TGF-β signaling in pan-cancer. TGF-β-associated extracellular matrix genes link CAFs to immune evasion and immunotherapy failure (57). Therefore, *YAP1* may be involved in the function of CAF through TGF-β signaling. We found that YAP1 inhibitor verteporfin reduced smad2 expression in CRC cell lines, suggesting a downregulation of TGF-β signaling (**Figure 8**). The role of *YAP1* in the infiltration and activity of T follicular helper cells, NKT cells, and activated NK cells remains to be investigated. Another interesting result in this study is that *YAP1* expression was negatively correlated with infiltration of CD8+ T cells, but positively correlated with infiltration of resting CD4+ memory T cells in most cancer types. This might not be due to the direct relationship between these two cells. *YAP1* expression was also negatively correlated with infiltration of activated CD4+ memory T cells, suggesting that *YAP1* may impede the activation of memory CD4+ T cells. CD4+ T cells are required for survival of CD8+ T cells during both primary and memory recall responses (119). Therefore, the possible relationship is that *YAP1* may impair the activation of memory CD4+ T cells, and impede the survival of CD8 T cells. Further experimental studies on CD4+ T cells will be conducted in the near future to verify the role of *YAP1* in memory CD4+ T-cell activation.

Further analyses showed that *YAP1* expression was positively correlated to a wide range of immune checkpoints, especially in PAAD, PCPG, and PRAD, suggesting that *YAP1* is a potential new drug target for anti-cancer immunotherapy in these cancer types. In PAAD, PRAD, and LGG, *YAP1* expression was positively correlated with the expression of *CTLA-4, TIM-3 (HAVCR2)*, and *PD-1 (PDCD1)*, which are key checkpoint regulators that suppress immune reaction. In most cancer types, *CD274 (PD-L1), NRP1*, and *TNFSF15* were positively correlated with *YAP1* expression. *YAP1* has been reported to induce *PD-L1* expression (115). Apart from *PD-L1*, *YAP1* might also regulate the expression of other immune checkpoints. The relationship between these immune checkpoints and *YAP1* and the role of their interactions in TME regulation remain to be investigated. These checkpoint regulators may play key roles in *YAP1*-induced immunosuppressive TME. In PAAD, PRAD, and PCPG, *YAP1* expression was highly correlated with the expression of most immune checkpoint genes in our result. Among them, PAAD has been reported to be characterized by the immunosuppressive microenvironment, suggesting a role for *YAP1* in PAAD development (102).

Methylation of DNA cytosine bases leads to the inaccessibility of DNA regulatory elements to their transcription factors through a number of mechanisms, leading to the gene transcription shutdown. We found that *YAP1* expression was negatively regulated by its DNA methylation in the 5’UTR. Cellular RNAs are naturally decorated with a variety of chemical modifications, which affect the mRNA stability and translation. In our study, extensive modification “effectors,” including enzymes of “writers” and “erasers” that alter the modification level and binding proteins of “readers” that recognize the chemical marks, were positively correlated with *YAP1* mRNA level. Further research should be conducted to study whether aberrant DNA methylation and RNA modifications of *YAP1* are involved in cancer development and how they work.

FDA-approved chemotherapeutic drugs that are capable of inhibiting DNA synthesis, including teniposide, dacarbazine, doxorubicin, and triethylenemelamine, and inhibitors of hypoxia-inducible factor, MCL-1, ribonucleotide reductase, and FASN in clinical trials are potential drugs to treat cancer patients with high *YAP1* expression. Among them, the cytotoxicity effect of AZD2858 and varlitinib was partially attributed to YAP1 activity.

## Conclusion

*YAP1* was aberrantly expressed in various cancer types and regulated by its DNA methylation and post-transcriptional modifications. High expression of *YAP1* was associated with poor survival outcomes in ACC, BLCA, LGG, LUAD, and PAAD. *YAP1* may promote tumor progression through immunosuppression, particularly by suppressing the infiltration of CD8+ T lymphocytes, CD4+ Th1 cells, T follicular helper cells, NKT cells, and activated NK cells, as well as recruiting MDSCs and CAFs in pan-cancer. The *YAP1*-promoting tumor activity is probably attributed to the activation of TGF-β, Hedgehog, or KRAS signaling pathways. AZD2858 and varlitinib in clinical trials might be effective in cancer patients with high *YAP1* expression.

## Data availability statement

The datasets generated during and/or analysed during the current study are available from the corresponding author on reasonable request.

## Author contributions

XH contributed to designing the article structure, writing—original draft, editing the figures and funding acquisition. YRZ contributed to performing experiments, editing the figures, and writing—review. HY contributed to performing experiments. YYZ and XS contributed to formal analysis. QL contributed to funding acquisition. YW contributed to supervision, funding acquisition, and project administration. All authors contributed to the article and approved the submitted version.

## Funding

This work was supported by the National Natural Science Foundation of China (81904130, 82122075, and 82074232), the Project of Shanghai Science and Technology Committee (19YF1449900), the Shanghai Frontier Research Base of Disease and Syndrome Biology of Inflammatory Cancer Transformation (2021KJ03–12), the “Shu Gguang” project supported by Shanghai Municipal Education Commission and Shanghai Education Development Foundation (21SG43), the Clinical Research Plan of SHDC (SHDC2020CR4043), and the Shanghai Youth Talent Support Program.

## Conflict of interest

The authors declare that the research was conducted in the absence of any commercial or financial relationships that could be construed as a potential conflict of interest.

## Publisher’s note

All claims expressed in this article are solely those of the authors and do not necessarily represent those of their affiliated organizations, or those of the publisher, the editors and the reviewers. Any product that may be evaluated in this article, or claim that may be made by its manufacturer, is not guaranteed or endorsed by the publisher.

## Supplementary material

Supplementary Figure 1 Transcriptional level of *TEAD1*, *TEAD3* and *TEAD4* in normal and cancer tissues according to RNA-sequence data from TCGA. *****p* < 0.0001, ****p* < 0.001, ***p* < 0.01, **p* < 0.05, ns *p* > 0.05.

## Glossary

**Table:**

ACC	adrenocortical carcinoma
BLCA	bladder urothelial carcinoma
BRCA	breast invasive carcinoma
CESC	cervical squamous cell carcinoma and endocervical adenocarcinoma
CHOL	cholangiocarcinoma
CI	confidence interval
CNTL	controls
COAD	colon adenocarcinoma
DFI	disease-free interval
DLBC	lymphoid neoplasm diffuse large B-cell lymphoma
DSS	disease-specific survival
ESCA	esophageal carcinoma
GBM	glioblastoma multiforme
GSEA	gene set enrichment analysis
HNSC	head and neck squamous cell carcinoma
HR	hazard ratio
IC50	half-maximal inhibitory concentration
KICH	kidney chromophobe
KIRC	kidney renal clear cell carcinoma
KIRP	kidney renal papillary cell carcinoma
LAML	acute myeloid leukemia
LCML	chronic myelogenous leukemia
LGG	brain lower-grade glioma
LIHC	liver hepatocellular carcinoma
LUAD	lung adenocarcinoma
LUSC	lung squamous cell carcinoma
MDSC	myeloid-derived suppressor cells
MESO	mesothelioma
MISC	miscellaneous
MSI	microsatellite instability
NES	normalized enrichment score
NK	natural killer
OS	overall survival
OV	ovarian serous cystadenocarcinoma
PAAD	pancreatic adenocarcinoma
PCPG	pheochromocytoma and paraganglioma
PFI	progression-free interval
PRAD	prostate adenocarcinoma
PVDF	polyvinylidene fluoride
READ	rectum adenocarcinoma
SARC	sarcoma
SKCM	skin cutaneous melanoma
STAD	stomach adenocarcinoma
TBST	Tris-buffered saline with Tween
TGCA	The Cancer Genome Atlas
TGCT	testicular germ cell tumor
THCA	thyroid carcinoma
THYM	thymoma
TMB	tumor gene mutation burden
UCEC	uterine corpus endometrial carcinoma
UCS	uterine carcinosarcoma
UVM	uveal melanoma
