# Supplementary figures and images for: The role of YAP1 in survival prediction, immune modulation, and drug response: A pan-cancer perspective
Source: Front Immunol. 2022 Nov 21;13:1012173. doi: 10.3389/fimmu.2022.1012173 (PMC9719955; doi:10.3389/fimmu.2022.1012173)

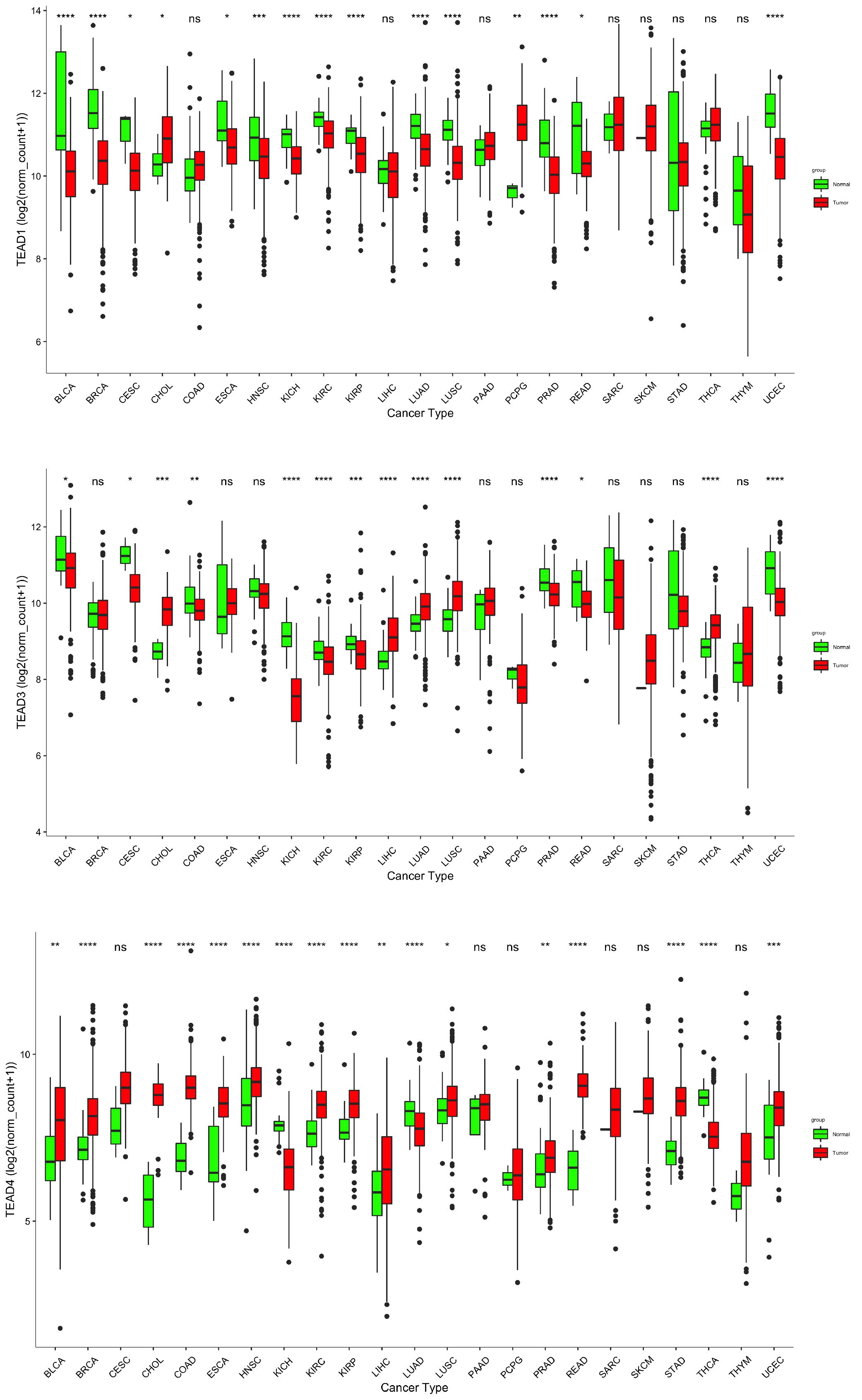

Supplement: Supplementary Figure 1 — Transcriptional level of TEAD1, TEAD3 and TEAD4 in normal and cancer tissues according to RNA-sequence data from TCGA. ****p < 0.0001, ***p < 0.001, **p < 0.01, *p < 0.05, ns p > 0.05. [file Image_1.jpeg]
